# Items

**Published:** 1887-02

**Authors:** 


### ITEMS.

The publication of the *Neurological Review* has been indefinitely suspended in consequence of the impaired health of its editor Professor J. S. Jewell.

The *American Journal of Biology*, edited by Dr. H. D. Valin, has recently been started in this city.

Another new medical periodical, called *The Medical Standard*, has recently made its appearance in this city. The name of the editor is not given, nor does it announce how frequently it will appear. Its publishers are J. P. Engelhard & Co. It is very neatly issued, on good paper, and with exceptional typographical accuracy.

A new dental journal, called *The Dental Review*, has been added to the list of recent publications in this city. Its editor is not announced. It is published by W. T. Keener.

Dr. N. S. Davis, on the 20th of January, completed the semi-centennial of his entry into the medical profession, and on that occasion he was the recipient of quite an ovation from the students of the Chicago Medical College, who assembled in the college, and, in the presence of many mutual friends, presented to their respected teacher and their firm friend, as a token of their appreciation of the man and his manifold works, a beautiful set of library furniture, with an inscription commemorating the occasion. An appropriate address of presentation was made, to which Professor Davis replied in terms which showed that he appreciated both the motive and the gift, and pointed the way to others who are preparing to enter the profession to do good to the world; to make their fellow-men better and happier, and to earn the rewards of a well-spent life, such as he himself must now be enjoying.

